# A Clinical-Epidemiological and Geospatial Study of Tuberculosis in a Neglected Area in the Amazonian Region Highlights the Urgent Need for Control Measures

**DOI:** 10.3390/ijerph18031335

**Published:** 2021-02-02

**Authors:** Cristal Ribeiro Mesquita, Emilyn Costa Conceição, Lúcia Helena Martins Tavares Monteiro, Odinea Maria da Silva, Luana Nepomuceno Gondim Costa Lima, Rafael Aleixo Coelho de Oliveira, Artemir Coelho de Brito, Ricardo José de Paula Souza e Guimarães, Karla Valéria Batista Lima

**Affiliations:** 1 Programa de Pós-Graduação em Biologia Parasitária na Amazônia, Universidade do Estado do Pará e Instituto Evandro Chagas, Tv. Perebebui, 2623, Belem 66087-662, Brazil; Luanalima@iec.gov.br; 2 Programa de Pós-Graduação em Epidemiologia e Vigilância em Saúde, Instituto Evandro Chagas, BR 316, km 7, Ananindeua 67000-000, Brazil; ricardojpsg@gmail.com; 3 Seção de Bacteriologia e Micologia, Instituto Evandro Chagas, BR 316, km 7, Ananindeua 67000-000, Brazil; 4 Programa de Pós-Graduação em Pesquisa Clínica em Doenças Infecciosas do Instituto Nacional de Infectologia Evandro Chagas, Fundação Oswaldo Cruz, Av. Brasil, 4365, Rio de Janeiro 21040-900, Brazil; emilyncosta@gmail.com; 5 Secretaria de Saúde do Estado do Pará-SESPA, Av. João Paulo II, 602, Belem 66087-048, Brazil; Tuberculose.para@gmail.com (L.H.M.T.M.); tuberculose.para@sespa.pa.gov.br (O.M.d.S.); 6 Programa de Pós-Graduação em Saúde, Ambiente e Sociedade, Universidade Federal do Pará, Rua Augusto Corrêa, Belem 66075-110, Brazil; artemir.brito@saude.gov.br; 7 Coordenação Geral de Vigilância das Doenças de Transmissão Respiratória de Condições Crônicas, Av. Brasil, 4365, Rio de Janeiro 21040-900, Brazil; aleixorafaelll@gmail.com

**Keywords:** tuberculosis, epidemiology, geospatial distribution, state of Pará, Brazil

## Abstract

Tuberculosis (TB) is an infectious communicable disease, which despite global efforts, still needs special attention in regions with difficult access. This study aims to describe the spatial and epidemiological trends of TB incidences from 2013 to 2018 in Marajó Island, the Amazonian region, Pará, Brazil. We have obtained secondary data from the Brazilian TB databases and performed geospatial and statistical analyses on the data for new TB cases, relapses, and re-admissions. From 2013 to 2018, 749 new cases were reported, in which the diagnostics (culture) was not performed for 652 (87.2%) patient samples, the drug resistance test (DST) was performed for nine (1.2%) samples, and one (0.13%) was multidrug-resistant TB (MDR-TB). The rapid molecular testing (RMT) was performed on 40 (5.3%) patient samples, with results indicating that eight (20%) were susceptible to rifampicin and two (5%) were rifampicin resistant. Overall, the cure rate was 449 (66.7%), while relapses and re-admissions were 41 and 44, respectively. On the geospatial distribution, the municipality of Soure stands out with a high number of incidences, relapses, and re-admissions. Spatially, the eight MDR cases were randomly distributed. Our data highlight the urgent need for TB control measures in this region, by introducing the Xpert-Ultra^®^ MTB/RIF (Cepheid, Sunnyvale, CA, USA) and Ogawa-Kudoh.

## 1. Introduction

Despite the scientific advances in infectious diseases, tuberculosis (TB), an ancient disease caused by *Mycobacterium tuberculosis*, is still one of the top 10 causes of death worldwide and the leading cause of death from a single infectious agent (ranking above Human Immunodeficiency Virus/Acquired Immunodeficiency Syndrome (HIV/AIDS)) [1], requiring greater attention from the professionals involved in the control and guarantee of public health care [2,3].

The risks for TB are mainly: Aging-related weakness of the respiratory system, weakness of the immune system caused by the use of immunosuppressive and licit/illicit drugs, alcoholic behavior, diseases such as diabetes, HIV/AIDS [4], and among other comorbidities, new infectious disease caused by a coronavirus (COVID-19) [5].

In Brazil, TB is a communicable disease, designated as a compulsory notification in public and private services that provide assistance to the patient. In addition, it is notified weekly based on the suspicion or confirmation of the illness/disease to the Health Department of the Municipality of the patient’s service location [6].

The laboratorial TB diagnosis in Brazil includes microscopy, which is the most widely used primary technique for the investigation of acid-fast bacilli (AFB) by the Ziehl-Neelsen (ZN) method and by rapid molecular testing (RMT). This is followed by, culture, the standard method with high specificity and sensitivity. Concerning the case of pulmonary TB with a negative smear microscopy, the sputum culture can increase the bacteriological diagnosis of the disease up to 30% [3].

The most used culture media for mycobacteria isolation are egg-based solids, Löwenstein-Jensen (LJ), and Ogawa-Kudo. The drug susceptibility test (DST) is performed based on the culture, in which two are available in Brazil: The proportion method, which uses the solid medium with results available after 42 days of incubation (lowest cost), and the method which uses the liquid medium, with results available after five to 13 days [3].

Among the three high-burden country (HBC) lists for TB, TB/HIV, and MDR-TB defined by the World Health Organization (WHO) for the period 2016–2020, which account for 80% of TB incidences globally, Brazil belongs to the TB and TB/HIV lists, as well as the top 20 countries with a high TB burden. Regarding the WHO Region of the Americas, the TB incidence is estimated to increase after many years of decline, owing to an upward trend in Brazil during 2016–2018 [1].

To address this worldwide problem, the Global Strategy for the End of TB was proposed with global guidelines for its eradication by the year 2035 [7,8]. Hence, constant measures for controlling TB are required, such as the strengthening of public health policies, the improvement of information systems, treatment management, and the improvement of human resources [9,10].

In Brazil, 73,864 new TB cases were diagnosed in 2019 with an incidence rate of 35.0/100,000 inhabitants, which, compared to the incidence in 2010 (37.5/100,000), was observed to decrease by 2.5 new incidences. However, when compared to 2018 (34.8/100,000), there was a slight increase in the rate of incidence. Among the Brazilian states, Pará has one of the highest incidence indicators, which is 51 cases/100,000 inhabitants [11], with an increase compared to 2018 (40.7/100,000) [12].

The State Health Plan (2016–2019) of the State Secretariat of Public Health of the State of Pará (SESPA) has pointed out seven municipalities as priorities for the control of TB, which are distributed in the Metropolitan Health Region I (Ananindeua, Belém, Marituba); Tocantins (Abaetetuba); Caetés (Bragança); Metropolitana III (Castanhal); and Baixo Amazonas (Santarém) [13]. Large areas, such as Marajó Island, still do not have a proper description of TB cases frequency, which is necessary for performing an epidemiological data analysis (incidence and prevalence) at the state level, and therefore, within the scope of SESPA, can be considered a neglected area of the state.

Marajó Island is one of the most important regions of agricultural and fishery production in the State of Pará, with emphasis on its large cattle and buffalo production as well as its derivatives (milk, cheese, and leather). However, despite its importance, the Marajó region has intense economical contrasts, corresponding to one of the poorest areas in the state of Pará and Brazil, with a population of 180,048 people living in extreme poverty [14]. With the lowest Human Development Indexes (HDI) in the country, of the 10 worst municipalities in Pará, six of them are located at Marajó Island [15,16].

To understand the compulsory notification disease such as TB, spatial analysis techniques came as an important subsidy for strategies for the prevention and control of communicable diseases. This analysis consists of a set of tools necessary to describe Public Health information, related to location, time, and disease mapping, in addition to being able to carry out an assessment of high risk prone areas for a given disease [17].

In this context, this study aimed to describe the spatial and epidemiological trends of TB notifications from 2013 to 2018 including the analysis of drug-resistance (DR) in Marajó Island, Pará, Brazil.

## 2. Materials and Methods

This is an epidemiological, descriptive, retrospective study with a quantitative approach on TB data from Marajó Island, Pará, Brazil Marajó Archipelago, where the main island, also called Marajó, is formed by 16 municipalities divided into two microregions: Marajó (Afuá, Cachoeira do Arari, Chaves, Muaná, Ponta de Pedra, Salvaterra, Santa Cruz do Arari, São Sebastião da Boa Vista e Soure) and Breves (Anajás, Bagre, Breves, Curralinho, Gurupá, Melgaço e Portel), covering a total area of 68,000 km^2^, this object is of interest in this study [13]. The study population consisted of all new TB cases, relapses, and re-admissions notified and confirmed to the Notifiable Diseases Information System (Sistema de Informação de Agravos de Notificação—SINAN, http://sinan.saude.gov.br/sinan/login/login.jsf) by the Pará Department of Public Health (Secretaria de Estado de Saúde Pública do Pará—SESPA), from 2013 to 2018. A new TB case is defined when an individual has never undergone anti-TB therapy or who underwent treatment for less than 30 days [3].

To clearly describe the data interpretation, we highlight the definitions for re-admission and relapse. Re-admission represents the return to treatment after abandoning the treatment, for example, a patient who, after starting the treatment for TB, stopped attending the health unit for more than 30 consecutive days, after the due date for his return. While relapse is defined as a TB patient who was already declared cured with a completed treatment, but who has reported back with TB symptoms and was confirmed as sputum smear positive within 5 years. If the time exceeds 5 years, a “new TB case” is considered and the recommended treatment is the basic scheme [3].

The epidemiological and demographic data were collected from SINAN, and the inclusion criteria included new cases of TB, with the place of residence on Marajó Island. The DR-TB data were obtained from the SIT-TB (http://sitetb.saude.gov.br/) database. The age group standardization was adopted according to the data available on the Brazilian National Health System Information Technology Department (DATASUS). The state, municipal, micro-, and mesoregion limits were obtained through the Brazilian Institute of Geography and Statistics (Instituto Brasileiro de Geografia e Estatistica—IBGE, 2010) data.

To reduce the data analysis bias and to elaborate the Geographic Database (BDGeo), a manual curation was performed. Subsequently, the cases of spatial distribution by municipality were performed using the ArcGIS 10.5 software (Esri, Redlands, CA, USA) (https://www.arcgis.com/). For spatial analyses, the incidence coefficient (CD_TB) was used, which is the risk of TB occurrence in a given municipality, with the basis of the population data from the last demographic census in 2010, multiplied by 100,000.

The risk incidence coefficient represents the risk of TB occurrence in the determined municipality, established through four quartiles of incidence. From these quartiles, the limits of incidence were established (IBGE 2010 and SINAN). The results for new cases were classified into four categories: (1) Low-light green (from 0.1 to 19.5); (2) Medium-dark green (from 19.6 to 26.9); (3) High-orange (from >27 to 31.5); and (4) Very High—red (from >31.6). The cases of relapse were classified as: Low-light green (from 0.1 to 0.69); Medium-dark green (from 0.70 to 1.39); High-orange (from 1.40 to 2.57); and Very-High-red (from 2.58–5.07). The cases of re-admission were classified as: Low-light green (from 0.1 to 0.71); Medium-dark green (from 0.72 to 1.45); High-orange (from 1.46 to 2.26); and Very-High-red (from 2.27–7.97).

The Global HDI and Municipal (MHDI) data were obtained from the United Nations Development Program website (Programa das Nações Unidas para o Desenvolvimento—PNUD—https://www.br.undp.org/), based on the year 2010. The HDI and MHDI are summary measures of long-term progress in three basic dimensions of human development: Income, education, and health. The closer to number 1, the higher the state/municipality development index [18].

The statistical analysis aimed to assess whether the data found in the study converge or differ to any trend. It was used as the standard descriptive analysis for absolute frequency and percentage analysis, and the application of statistical tests: Pearson’s non-parametric Chi-square test (Wilks’ G^2^) for independence, adopting a significance level of *p*-value < 0.05. For the test interpretation, we assumed a null hypothesis (H_0_) when the observed frequencies occurred in the same proportion for the different groups, and as an alternative hypothesis (H_a_) when the observed frequencies differed significantly for the different groups.

Based on that, a decision is made since the computed *p*-value is less than the significance level of α = 0.05, the H_0_ should be rejected, and H_a_ accepted. A linear trend model was also used to calculate the trend of new cases in a spatio-chronological manner. In this way, the collected data were tabulated, interpreted, processed, and analyzed using computing resources, through processing in the Microsoft Office Excel software, Statistic Package for Social Sciences (SPSS) version 22.0, and Epi Info 7.2, all in a Windows 7 environment.

The Spearman’s rank correlation coefficient (rho) was used to analyze the correlation between MHDI and different types of TB (new cases, relapses, and re-admissions) using the R software (https://www.r-project.org/).

Regarding the ethical aspects, our study was conducted according to the Resolution 466/2012 of the National Health Council/Health Ministry. Since the research used secondary data, the Informed Consent Term was not necessary. This study was then approved by the Research Ethics Committee of the Universidade do Estado do Pará, under number 3.705.199.

## 3. Results

### 3.1. Analysis of the Trend of New Cases

**Figure 1:**
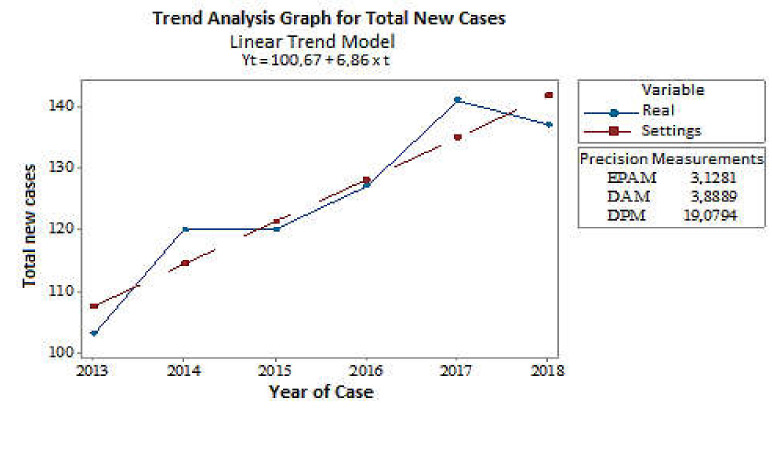
A temporal series for analyzing the trend of new cases of human tuberculosis on the Marajó Island, Pará-Brazil, from 2013 to 2018. The variable “Real” means absolute data, while the variable “Settings” means linear model. Source: Research protocol (2020).

Between 2013 and 2018, there were 749 new TB cases registered in Marajó Island (2013-103; 2014-120; 2015-120; 2016-127; 2017-141; 2018-138), with an average of 125 cases/year (µ = 124.7) and a standard deviation of 14 cases (σ = 13.7). Figure 1 shows that the series of new cases has an increasing trend, since the equation coefficient is positive (b = +6.86). This model reveals a trend of cases for the coming years, in this case, there is an increasing trend. Additionally, the linear regression analysis has demonstrated that as time passes, it tends to increase the number of cases (*p-*value < 0.001).

### 3.2. Description of Clinical and Epidemiological Data

Table 1 shows that the majority of new TB cases identified in Marajó Island are in the age range between 20 and 39 (334; 45%); mean of 37 years (µ = 37.29 ± 18.59); a minimum of 0 years (less than 1 year old) and a maximum of 99 years old; most are male (487; 65%); and the “Brown” (“parda”) race is predominant with 611 (82%) cases, 201 (27%) with schooling between 1st and 4th grade of elementary school.

Regarding the clinical TB characteristics, most were diagnosed as pulmonary (686; 92%), followed by extrapulmonary (43; 5.8%), and mixed disease (19; 2%). Among the cases of extrapulmonary and/or mixed form, the most reported were: Pleural (26; 42%); ganglionic (13; 21%); miliary (11; 18%), brain meningoencephalitis (3; 5%); and laryngeal, bony, disseminated, intestinal, axillary accessory breast with a single case in each one.

We observed that only 16 (2.1%) new cases presented associated diseases and conditions of the mental illness type, while 42 (5.6%) have other associated diseases and conditions. The main disease that affects new TB cases on Marajó Island is arterial hypertension (13; 1.7%) (Table 2).

### 3.3. Description of New Tuberculosis Cases According to the Tuberculosis Culture Results, HIV Diagnosis, and Closure Situation

It was observed that only 64 (8.6%) new cases had a positive diagnosis by culture and 40 (5.3%) had a positive diagnosis for HIV. Most of the new cases presented a cure situation for TB (499; 66.7%). There were 23 (3.1%) deaths from TB and 27 (3.6%) deaths from other causes (Table 3).

### 3.4. Analysis of MDR-TB Cases: Relapse and Re-Admission

During the period of study, eight cases of TB-DR were reported, of which seven were MDR-TB (Table 4). Moreover, seven were reported as acquired resistance and one as primary resistance. Six cases were male, aged 20 to 47 years (µ = 32.14 ± 9.85), residing in Cachoeira do Ararí (two cases), Ponta de Pedras, Santa Cruz do Ararí, Portel and Soure, and two females, aged 24 and 25, both residents in Breves.

Regarding the cases of relapse, 41 cases were reported on Marajó Island in the period 2013–2018. Most cases were male (23; 56%), adult age group (31; 76%), mixed race (35; 85%), with incomplete elementary schooling (11; 27%).

Of the 41 relapse cases, 40 were not tested by RMT or DST (40; 98%). In the trend analysis of cases, there is an irregular trend in the raw data, from 2016 to 2017 there was a sharp drop, for 2018 there is a small increase in cases, but in the adjustable trend there is a drop in cases. Regarding the 44 cases of re-admission reported, the majority were male (26; 59%); adult age group (36; 82%); mixed race (37; 84%); with incomplete elementary schooling (10; 23%). The RMT and DST were also not performed either for relapse or for re-admission cases. When analyzing these case trends, we observed a tendency to decrease both relapse and re-admission cases.

### 3.5. Geospatial Distribution of Cases by Municipality

**Figure 2:**
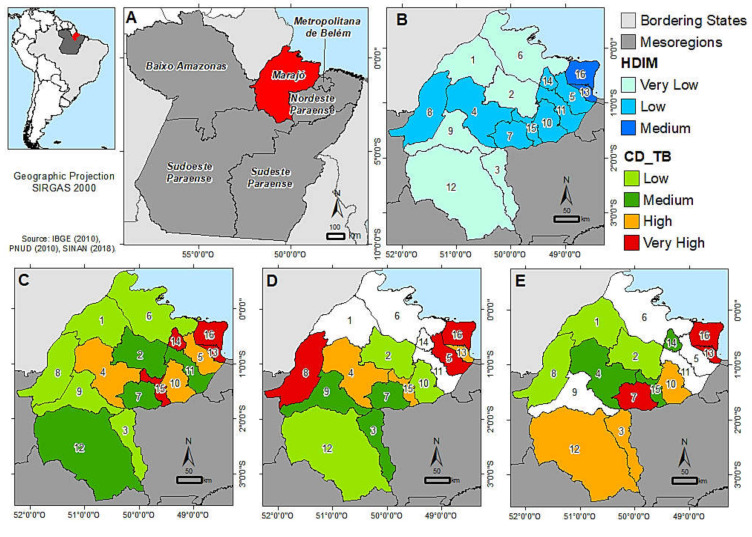
(**A**) Mesoregions of Pará; (**B**) Municipal Human Development Index (HDIM); (**C**) new cases; (**D**) relapse; and (**E**) re-admission of tuberculosis cases by the municipality of Marajó Island, 2013–2018. The limits of the color scales of incidence coefficient (CD_TB) were established by quartiles according to each incidence.

Figure 2C shows a grouped spatial pattern, from all the municipalities of Marajó Island, bringing together all new cases (2013–2018). There were four municipalities (Salvaterra—13, Santa Cruz do Arari—14, São Sebastião da Boa Vista—15 and Soure—16) with “very high” TB incidence. When analyzing the gross cases of TB, there are places where the cases exceed the absolute number of these municipalities. However, upon analyzing the total area, higher cases are observed per inhabitant in these five municipalities. Soure (16) and Salvaterra (13) are the cities with the highest incidence rates, 55.07 and 41.28/100,000 inhabitants, respectively are geographically neighboring and are the cities that receive more tourists due to the privileged location. Likewise, the neighboring municipality of Salvaterra, Cachoeira do Arari (5) had an incidence of 27.71 cases/100,000 inhabitants.

The municipalities of Afuá (1), Bagre (3), Chaves (6), Gurupá (8), and Melgaço (9) showed the lowest incidence of TB. Afuá alone showed 3.32 cases/100,000 inhabitants. When analyzing the municipality with the largest geographical area (Breves) (12), the absolute number of cases was 173 cases, but due to the population number, it was classified as a medium incidence rate.

We can observe the cases of relapse TB (or recurrence) (Figure 2D), which were not found in four cities (Afuá, Chaves, Ponta de Pedras, and Santa Cruz do Arari), while three municipalities stand out with a number of cases of recurrence in the interval of 2013–2018 (Cachoeira do Arari—4.076/100,000 inhabitants, Soure—5.0772/100,000 inhabitants, Gurupá—2.867/100,000 inhabitants), the city of Salvaterra (2.477/100,000 inhabitants), Breves (1.436/100,000 inhabitants), and São Sebastião da Boa Vista (1.455/100,000 inhabitants) are classified as having a high recurrence rate.

The re-admission cases are shown in Figure 2E. The municipality of Chaves remains without any cases and adds Cachoeira do Arari, Melgaço, and Ponta de Pedras in this classification. Soure (7.970/100,000 inhabitants), Salvaterra (4.087/100,000 inhabitants), and Curralinho (2.335/100,000 inhabitants) are classified with a high prevalence of re-admission cases.

Upon analyzing the HDI and MHDI in the municipalities, it can be seen in Figure 2B that the average for Brazil, in 2010, is 0.755 (high MHDI-75th position), with the state of Pará, 0.646, below 0.109 of the Brazilian average, occupying the 24th position of 27 Brazilian states (PNUD, 2010). No municipality on the Marajó Island is above or has the same average as Pará, the cities that are closest are: Soure (0.615) and Salvaterra (0.608). The municipality of Melgaço occupies the last position (5565th) in the MHDI ranking of Brazilian cities, in the company of Chaves (5560th) and Bagre (5558th), composing the list of the ten worst Brazilian MHDI. All municipalities on the Island are below the 5000th, except for Soure (3796th) and Salvaterra (3957th).

The following correlations were found using Spearman’s correlation coefficient (rho): MHDI and relapses (rho = 0.3288979, *p-*value = 0.2136); MHDI and re-admissions (rho = 0.3303794, *p-*value = 0.2114); MHDI and new cases (rho = 0.8147059, *p*-value = 0.0001719). Therefore, only a significant correlation was obtained between the MHDI and new TB cases in Marajó Island.

## 4. Discussion

More than a century has passed since the TB agent *M. tuberculosis* was described by Robert Koch in 1882. However, the TB epidemic is still challenging in many aspects, in which the intervention with an early diagnosis to intercept the chain transmission, the treatment adherence, the evolution and spread of multidrug resistant TB (MDR-TB) strains, and the underreporting of TB cases, is highlighted. Although the last one has also been found in several countries, it is known that underreporting is mainly due to the lack of knowledge of notifiable diseases and problems in the notification flow carried out by health professionals [19,20].

The TB incidence rate in Marajó Island from 2013 to 2018 ranges from 55.07 to 32/100,000 inhabitants for each municipality, which needs to change compared to the international goal for the period 2020–2035 proposing a reduction of 20% in the incidence of TB globally [21]. In our study, however, the average number of TB cases was 125 cases/year, demonstrating an increasing trend during the time period analyzed, unlike the general trend in other regions of Brazil [22,23] and contrary to the global trend that the incidence has decreased, on average, 1.5% per year since 2000 [21].

Possibly the trend of increasing the number of new cases is due to failures in the early detection of cases and greater transmission of TB. However, in recent years, there has been an intensification of TB monitoring in the region, which may be associated with the increase in the number of diagnoses. The number of notifications is expected to increase even further with the search for contacts and the availability of the PPD or IGRA and radiography service to detect latent cases. Other studies developed in the state capital have already pointed to the need for greater surveillance of TB and the use of strategies to control transmission [24]. In another study, there were oscillations in the number of contaminations by TB, but there was no significant reduction in cases during the years of study [23].

From a sociodemographic perspective, it is important to note that the majority of cases occurred in adults (20–39 years old), economically active age groups, followed by the elderly population (>60 years old) and, mostly male, corroborated with other studies [22,23,25]. Males are more exposed due to their historical and social activities to risk factors, also associated with seeking health services less frequently [25]. The predominant race was “Brown” (“*parda*”), the most predominant race in Brazil [23,26]. Regarding education, the TB prevalence is inversely proportional to the level of education [23,25], since there is more TB among those with an incomplete primary education than in the high level of education. This can be a challenge for TB control, while developing educational actions for this disease prevention.

The most prevalent clinical form was pulmonary TB, being the infectious form of the disease, through the inhalation of contaminated droplets expelled by an infected person [23], corroborated with other studies [22,23,25,27,28]. Due to the fact that the notification forms are not filled properly, which is a limitation in this study, also leads to the difficulty in evaluating and planning strategies for TB prevention and control. This can be observed in the analysis of diseases and other associated grievances, in which 27.1% of the cases, ignored (13.9%) and no information of the forms (13.2%) were not filled out, and among the 42 cases with associated diseases, the systemic arterial hypertension was the most frequent disease.

The Ministry of Health recommends that individuals diagnosed with TB should be tested for HIV, as TB-HIV co-infection leads to a worse prognosis [29]. Nevertheless, in our study, 39.4% of the cases were not tested for HIV, which is a failure in the screening protocol, similar to the other study [23].

This fact may be related to the patients’ place of residence, in the capital of Pará only one case has not been reported for HIV serology, since it is a simple diagnostic test with low cost and available in several health units. A reluctance has been observed towards the HIV serology test by the island residents, as they report that a positive test result could be revealed among laboratory professionals, patients’ acquaintances, and family members [27].

The cases of relapse and re-admission are important to be analyzed, since they may have a direct relationship on the MDR-TB cases. Relapse is due to the fact that bacilli resistance, the resistance to drugs used, or non-adherence to the treatment are factors that deserve to be well studied to identify the variables which may be contributing to this event [30].

The cause of bacillary reactivation has not yet been defined. It is suspected that 90% of the infected individuals remain in a latent state and only 5% of these individuals have healed and presented reactivation. Re-admission cases after treatment abandonment is a determining factor for the emergence of MDR-TB cases [31].

The same error occurs while performing the culture test and the diagnostic test, which is considered the TB standard [32,33], in which 87.2% were not performed. Not performing the culture analysis is a “bad culture”, which can lead to a blind treatment especially for MDR-TB cases or for pulmonary diseases caused by non-tuberculous mycobacterias (NTM) (mycobacteriosis), such as *Mycobacterium kansasii*, which are undistinguished from TB clinically or through microscopy. In both scenarios, the outcome is not positive.

Regarding the outcomes, the status “cure” was the most predominant, in accordance with the previous studies [22,23] and followed by the treatment abandonment (11.5%), which is higher than the WHO recommendations (<5%) and can be explained by the following: Access to treatment, difficulty in obtaining information, lack of medication, absence of active search, and characteristics related to the health unit access including expenses from transportation and the distance from home [26].

Due to the low cure outcome in this region, it is necessary for the local TB control service to intensify the search for cases of abandonment and treatment failure. In addition, to improve the diagnostic strategies for detecting MDR-TB using the RMT for TB and the rifampicin resistance detection endorsed by WHO [34] Xpert-Ultra^®^ MTB/RIF (Cepheid, Sunnyvale, CA, USA), which is available in Brazil [35]. Moreover, by performing culture, the gold standard (reference technique) for TB diagnosis, which also allows the detection of NTM. In this aspect, we highlight that lung damage resulting from a previous TB infection reduces the normal elimination of pathogens, causing a proportion of TB patients to be at an increased risk for NTM infection. Both MDR-TB and MNT cases require a different treatment regimen than the regimen I used for primary TB. The Marajó Island does not yet have the recommended Xpert-Ultra^®^ MTB/RIF (Cepheid, Sunnyvale, CA, USA). Based on this study, we suggest the implementation of the RMT in the municipalities of Salvaterra or Soure.

In view of the difficulties of accessing most villages, the recommended culture medium should preferably be Ogawa-Kudoh, which can be used by laboratories that do not have all the recommended equipment (especially a refrigerated centrifuge) for other methods. It is economical and sensitive enough to ensure that the culture contributes to confirming the diagnosis of pulmonary TB, in suspected cases with negative sputum smear microscopy and useful for recovering sputum bacilli from bacilliferous patients requiring sensitivity testing [3].

A recent study isolated *M. tuberculosis* var. *bovis* with unique genotypes from cattle and buffaloes in slaughterhouses from Marajó Island [36], which was later fully characterized by whole-genome sequencing (WGS). In addition, compared to the national and international WGS data, this study demonstrates that these strains were not recently introduced in this region. This is possible due to the geographic isolation of the region, including difficulties in accessing inter-municipalities [37]. Predominant clades are also possible among human TB cases, but genetic studies should be developed.

When evaluating the geospatial patterns, the TB incidence rate was higher in the two most touristic cities on the Island: Soure and Salvaterra, which also corresponded to the highest levels of MHDI (0.615/0.608), although they are still below the state average (0.646). The Marajó Island is part of the State of Pará, which is a region still neglected in terms of basic life issues and became a key point for the accentuated transmission of neglected diseases, such as bovine TB [16,37].

This association between cities with low infrastructures, low levels of MHDI, and high rates of TB incidence demonstrates the vulnerability of the population and difficulty in accessing health services for diagnosing the TB treatment [38]. This difficulty leads to a greater chance for an infected person to transmit to other people, especially in places with geographical barriers such as islands [26]. These politico-economic-geographical barriers also lead to treatment difficulties in conjunction with variables related to the adverse effects of the medication and the performance of the health service.

In this context, we suggest the implementation of Xpert-Ultra^®^ MTB/RIF (Cepheid, Sunnyvale, CA, USA) and Ogawa-Kudoh in this region, which could improve the TB surveillance in Marajó Island by searching for TB active individuals and avoiding the samples shipment to the capital (Belém, Pará) by the Maritimes and land transport, as well as decreasing the time of diagnosis. Additionally, it is necessary to perform professional training among the health care team at the basic level, while ratifying the importance of properly filling the TB notification form, as well as improving the quality of studies and reports based on secondary data.

## 5. Conclusions

This study describes the TB situation in an Amazonian Region of Brazil, at Marajó Island-Pará. Based on the notifications from 2013 to 2018, this region needs urgent attention from the TB surveillance program to improve the TB diagnosis and a proper collection of epidemiological data. Within the Marajó Island, we highlighted the municipality of Soure which stands out with a high TB incidence, relapse, and re-admission. We also demonstrated that among the DR, there was not any cluster for MDR-TB cases. However, our data highlight the urgent need for TB control measures in this region for identifying active and resistant TB, which could be improved by the introduction of an RMT, such as Xpert-Ultra^®^ MTB/RIF, as well as a basic conventional test, such as culture by the Ogawa-Kudoh method.

## Figures and Tables

**Table 1:** Distribution of new human tuberculosis cases in Marajó Island, Pará-Brazil, according to epidemiological data.

Epidemiological Characterization (New Cases)	Number	%	*p-*Value ^(1)^
Age Range (year)			
From <1	09	1%	<0.001 *
From 1 to 19	106	14%	
From 20 to 39	334	45%	
From 40 to 59	190	25%	
From ≥60	110	15%	
Total	749	100%	
Sex			
Female	262	35%	<0.001 *
Male	487	65%	
Total	749	100%	
Total	749	100%	<0.001 *
Race/Skin Color			
White	65	9%	<0.001 *
Black	57	8%	
“Brown” (“Parda”)	611	82%	
Yellow	5	1%	
Indigenous	1	0%	
Ignored	10	1%	
Total	749	100%	
Education			
Illiterate	76	10%	0.021 *
Incomplete elementary school (1th to 4th series)	201	27%	
Complete elementary school (old “1 degree”)—4th Series	36	5%	
Incomplete elementary school (old “1 degree”)—5th to 8th Series	124	17%	
Complete elementary school (old “1 degree”)	35	5%	
Incomplete high school (old “2 degree”)	59	8%	
Complete high school (old “2 degree”)	53	7%	
Incomplete higher education	7	1%	
Complete higher education	12	2%	
Ignored	123	16%	
Not applicable **	23	3%	
Total	749	100%	
Clinical Characteristics			
Pulmonary	686	92%	
Extrapulmonary	43	5.8%	
Pulmonary + Extrapulmonary	19	2%	<0.001 *
Ignored	1	0.2%	
Total	749	100%	

Note: The results are based on non-empty rows and columns in each innermost suitable. Source: Research protocol (2020). ^(1)^ Pearson’s Chi-square test (Wilks’ G^2^) for independence (*p*-value < 0.05). * Significant values; NS: Non-significant values. ** Unfilled item.

**Table 2:** Distribution of new cases of human tuberculosis on the Marajó Island, Pará-Brazil, according to diseases and conditions.

Diseases (New Cases)		Number	%	*p-*Value ^(1)^
Diseases and Associated Grievances (Mental Disease)	1. Yes	16	2.1%	0.001 *
	2. No	664	88.8%	
	9. Ignored	32	4.3%	
	No information	36	4.8%	
Diseases and Associated Grievances (Others)	1. Yes	42	5.6%	0.031 *
	2. No	503	67.2%	
	9. Ignored	104	13.9%	
	No information	99	13.2%	
Diseases and Associated Grievances	Not applied ^(2)^	707	94.5%	0.031 *
	Asma	1	0.1%	
	Amygdala Cancer	1	0.1%	
	Breast cancer	1	0.1%	
	Cardiopathy	2	0.3%	
	TB contact	1	0.1%	
	Insanity	1	0.1%	
	Depression	1	0.1%	
	Illicit drugs	3	0.4%	
	Epilepsy	1	0.1%	
	Ex-Smoker	1	0.1%	
	Pulmonary Fibrosis	1	0.1%	
	Smoking	1	0.1%	
	HAS	13	1.7%	
	Hepatitis	3	0.4%	
	LTA	1	0.1%	
	Pneumonia	1	0.1%	
	Chronic Kidney	1	0.1%	
	Syphilis	2	0.3%	
	Down Syndrome	2	0.3%	
	Smoking	2	0.3%	
	VDRL+	1	0.1%	

TB: Tuberculosis; HAS: Arterial hypertension; LTA: Cutaneous leishmaniasis; VDRL: Venereal Disease Research Laboratory. Note: Results are based on non-empty rows and columns in each innermost subtable. Source: Research protocol (2020). ^(1)^ Pearson’s chi-square test (Wilks’ G^2^) for independence (*p*-value < 0.05). ^(2)^ Patient without the associated disease. * Significant values; NS: Non-significant values.

**Table 3:** Distribution of new cases of human tuberculosis on the Marajó Island, Pará-Brazil, according to the result of the culture, HIV diagnosis, and the situation of closure.

Culture/HIV/Closing (New Cases)		Number	%	*p-*Value ^(1)^
Culture	1. Positive	64	8.6%	0.006 *
	2. Negative	17	2.3%	
	3. Waiting result	15	2.0%	
	4. No performed	652	87.2%	
HIV	1. Positive	40	5.3%	0.001 *
	2. Negative	384	51.3%	
	3. Waiting result	29	3.9%	
	4. No performed	295	39.4%	
Closing Situation	1. Cure	499	66.7%	<0.001 *
	10. Primary Abandonment	2	0.3%	
	2. Abandonment	86	11.5%	
	3. Death by TB	23	3.1%	
	4. Death from other causes	27	3.6%	
	5. Transferred	64	8.5%	
	6. Diagnostic Change	3	0.4%	
	7. Tuberculosis drug resistance	8	1.1%	
	8. Scheme Change	3	0.4%	
	9. Failure	2	0.3%	
	No information	31	4.1%	

Note: Results are based on non-empty rows and columns in each innermost suitable. Source: Research protocol (2020). ^(1)^ Pearson’s Chi-square test (Wilks’ G^2^) for independence (*p*-value < 0.05). * Significant values; NS: Non-significant values.

**Table 4:** First-line drug-resistant tuberculosis cases on Marajó-Pará Island and the closure situation.

Start Date of Treatment	Sex	Age	Municipality of Residence	Drug Resistance Profile	Type of Resistance	Pulmonary Type	Outcome	HIV Status
30 January 2013	M	47	Portel	MDR	Acquired	Bilateral cavity	Cured	No
6 August 2013	M	45	Santa Cruz do Arari	MDR	Acquired	Bilateral cavity	Failure	Yes
21 March 2016	F	24	Breves	MDR	Acquired	Unilateral c cavity	Failure	No
30 December 2016	M	29	Ponta de Pedras	MDR	Primary	Unilateral non-cavitary	Abandon	Yes
4 January 2017	F	25	Breves	MDR	Acquired	Unilateral cavity	Cured	No
1 December 2017	M	28	Cachoeira do Arari	Rifampicin	Acquired	Unilateral c cavity	Failure	No
29 March 2018	M	20	Soure	MDR	Acquired	Bilateral non-cavity	Cured	No
28 May 2018	M	28	Cachoeira do Arari	MDR	Acquired	Unilateral cavity	In treatment	No

M: Male; F: Female; HIV: Human immunodeficiency virus; MDR: Multidrug resistance. Note: The results are based on non-empty rows and columns in each innermost suitable. Source: Research protocol (2020).
